# A Promising Way to Overcome Temozolomide Resistance through Inhibition of Protein Neddylation in Glioblastoma Cell Lines

**DOI:** 10.3390/ijms24097929

**Published:** 2023-04-27

**Authors:** Barbara Brandt, Marica Németh, Gergely Berta, Máté Szünstein, Marija Heffer, Tibor A. Rauch, Marianna Pap

**Affiliations:** 1 Department of Medical Biology and Central Electron Microscopic Laboratory, Medical School, University of Pécs, 7624 Pécs, Hungary; barbara.brandt@aok.pte.hu (B.B.);; 2 Department of Ecology, Faculty of Sciences, University of Pécs, 7624 Pécs, Hungary; 3 Department of Medical Biology and Genetics, Faculty of Medicine, Josip Juraj Strossmayer University of Osijek, 31000 Osijek, Croatia; 4 Department of Biochemistry and Medical Chemistry, Medical School, University of Pécs, 7624 Pécs, Hungary

**Keywords:** glioblastoma multiforme, temozolomide, neddylation, combination treatment

## Abstract

There is no effective therapy for the lately increased incidence of glioblastoma multiforme (GBM)—the most common primary brain tumor characterized by a high degree of invasiveness and genetic heterogeneity. Currently, DNA alkylating agent temozolomide (TMZ) is the standard chemotherapy. Nevertheless, TMZ resistance is a major problem in the treatment of GBM due to numerous molecular mechanisms related to DNA damage repair, epigenetic alterations, cellular drug efflux, apoptosis-autophagy, and overactive protein neddylation. Low molecular weight inhibitors of NEDD8-activating enzyme (NAE), such as MLN4924, attenuate protein neddylation and present a promising low-toxicity anticancer agent. The aim of our study was to find an effective combination treatment with TMZ and MLN4924 in our TMZ-resistant GBM cell lines and study the effect of these combination treatments on different protein expressions such as O^6^-methylguanine methyltransferase (MGMT) and p53. The combination treatment successfully decreased cell viability and sensitized TMZ-resistant cells to TMZ, foreshadowing a new treatment strategy for GBM.

## 1. Introduction

The World Health Organization (WHO) has introduced an international grading scheme for the classification and diagnosis of gliomas [1]. The most aggressive WHO grade 4 glioblastoma multiforme (GBM) is also the most common primary malignant brain tumor in the central nervous system (CNS), accounting for 50% of all primary brain gliomas [2,3]. The prognosis for patients with GBM is very poor, with a median survival from diagnosis of about 15 months [4].

The standard therapy includes the surgical resection of the majority of the tumor mass followed by radiotherapy and chemotherapy [4]. Despite several international efforts, its treatment faces huge challenges, such as the presence of the blood–brain barrier (BBB) which prevents ~98% of small molecule drugs from entering the CNS [5], and the diffuse invasiveness of the tumor cells [6]; therefore, surgical excision does not prevent tumor recurrence or increase survival [7]. To increase the effectiveness of treatments, chemotherapeutic agents have been tested to improve the survival of patients, including DNA alkylating agents such as temozolomide (TMZ), bis-chloroethylnitrosourea (BCNU), and lomustine (CCNU) [8]. Of these agents, TMZ is the drug of choice for standard chemotherapy [8] since it was approved by the US Food and Drug Administration (FDA) in 2005 [9]. TMZ is a second-generation imidazotetrazine lipophilic prodrug that is able to cross the BBB and can therefore be administered orally [10]. The active metabolite 5-(3-methyltriazol-1-yl)imidazole-4-carboxamide (MTIC) [11] forms and further hydrolyzes to 5-amino-imidazole-4-carboxamide (AIC) and a methyldiazonium ion. The methyl group of the methyldiazonium ion is transferred to specific sites on the DNA, resulting in the formation of N^7^ methyl guanine (N^7^MG), N^3^ methyl adenine (N^3^MA), and O^6^ methyl guanine (O^6^MG) [9]. The formation of O^6^MG leads to its mismatch with thymine instead of cytosine [9]. These mismatches are recognized by the mismatch repair (MMR), which removes thymine from the undamaged DNA strand instead of removing the O^6^MG, therefore leading to additional thymidine incorporation during DNA synthesis [12]. These unrepairable DNA damages generate the formation of single- and double-stranded DNA breaks and trigger cell cycle arrest in the G2/M phase and apoptosis in glial cells [9].

TMZ resistance is a major problem in the treatment of GBM and it has been associated with numerous molecular mechanisms including DNA repair, multidrug transporters, epigenetic modifications, microRNAs, extracellular vesicles, and autophagy [9]. O^6^-methylguanine-DNA methyltransferase (MGMT) is generally considered to be the most important contributor to TMZ resistance [9]. MGMT is an evolutionarily conserved DNA repair enzyme that protects the cellular genome from the mutagenic effects of TMZ [10]. It maintains genomic stability through MMR; however, during TMZ treatment, MGMT is able to remove the methyl group from O^6^MG, therefore, reversing TMZ-induced DNA damage and reducing the efficiency of the treatment [9]. The alkyl group from O^6^MG is transferred to the MGMT’s own cysteine residue, restoring thereby normal guanine [10]. This reaction results in the inactivation of MGMT, leading to its degradation by the proteasome, so an enzyme can only remove one adduct, thus this repair mechanism depends on continuous MGMT protein expression [12]. The epigenetic status of MGMT, such as promoter methylation, histone modifications, and miRNA regulation of transcription levels is considered a marker of intrinsic resistance to TMZ [12]. The expression of MGMT is partly determined by the CpG methylation status of the promoter region of the MGMT gene—promoter hypermethylation leads to decreased expression of the MGMT protein and correlates with prolonged survival of GBM patients [9]. On the other hand, the unmethylated MGMT promoter is associated with increased MGMT protein expression, leading to resistance to TMZ [9]. Members of the ATP-binding cassette (ABC) protein transporter family play an important role in the development of chemoresistance through the transport of solutes, drugs, and xenobiotics across cell membranes [13,14]. ABC transporter proteins involved in GBM mostly belong to the ABCB, ABCC, and ABCG subfamilies [13]. ABCG2 (ATP binding cassette subfamily G member 2) overexpression enhances chemoresistance in GBM to several chemotherapeutic agents such as vincristine, topotecan, irinotecan, and TMZ [13]. Although ABCC1 is overexpressed in high-grade gliomas and is associated with multidrug resistance in cancer cells, the inhibition of ABCC1 has not led to TMZ sensitization in GBM [15].

Protein neddylation is a recently described reversible post-translational modification by which NEDD8 (neural precursor cell expressed, developmentally downregulated 8) protein is conjugated to a lysine residue of a targeted protein [16]. Just as in ubiquitination, this process requires a three-step enzymatic reaction, catalyzed by NEDD8-activating enzyme E1 (NAE1), NEDD8-conjugating enzyme E2s (UBC12/UBE2M or UBE2F), and substrate-specific NEDD8-E3 ligases [17]. The best-described neddylation substrates are the cullin subunits of Cullin-RING ligase (CRL), which functions as a ubiquitin ligase and controls the turnover of a number of proteins that play important roles in physiological and pathological conditions, including carcinogenesis [18]. Along with cullins, numerous proteins are substrates of neddylation such as p53, mouse double minute 2 (Mdm2), and epidermal growth factor receptor (EGFR); therefore, neddylation plays a role in various crucial biological processes as well as transcription, proteolysis, inflammatory responses, differentiation, signal transduction, and tumorigenesis [16]. Lately, experimental evidence proves that protein modification by neddylation is overactivated in numerous human cancers (lung cancer, intrahepatic cholangiocarcinoma, liver cancer, colorectal cancer, glioblastoma, nasopharyngeal carcinoma, esophageal squamous cell carcinoma) [17] due to the overexpression of NEDD8 and several neddylation enzymes [19]. Furthermore, overexpression of these enzymes is associated with disease progression; therefore, inhibition of protein neddylation became an attractive anticancer strategy [17]. In order to suppress neddylation, a drug called MLN4924 (also known as pevonedistat) was discovered by Millennium Pharmaceuticals [17]. MLN4924 is a small molecule NAE inhibitor and can bind to active sites of NAE, therefore, inhibiting the progress of the next enzyme reaction [19]. This inhibition results in suppressed cullin neddylation and causes the accumulation of CRL-substrates leading to DNA damage, cell cycle arrest, senescence, and apoptosis in different cancer species [19]. Moreover, increasing experimental evidence suggests that MLN4924 suppresses glioblastoma cell growth in vitro and in vivo [19]. According to preclinical studies, MLN4924 has potent antitumor activity against several solid tumors and hematological malignancies [17]. Moreover, in phase I/II clinical trials, MLN4924 showed good clinical efficiency and low toxicity [19]. Furthermore, preclinical studies proved that MLN4924 in combination with chemotherapy increased antitumor activity in solid tumor cell lines and xenograft models [17].

## 2. Results

### 2.1. Determination of the Methylation Status of the MGMT Promoter in GBM Cell Lines

**Figure 1:**
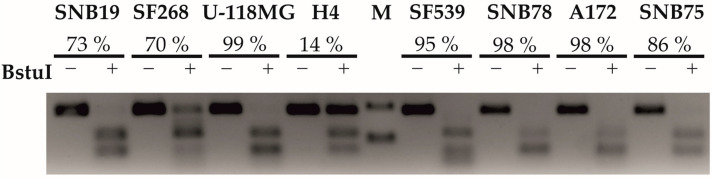
Methylation status of the MGMT promoter in GBM cell lines. Genomic DNA was extracted and treated with bisulfite; the MGMT promoter region was amplified by PCR, digested with BstUI, and visualized by agarose gel electrophoresis as described in Section 4. For semiquantitative evaluation, the percentage of cleaved (methylated) DNA was determined using ImageJ software (version 1.53t). M = molecular weight marker.

Resistance to chemotherapy is a major problem in the treatment of GBM. The methylation status of the MGMT promoter region is used as a marker for TMZ resistance, the hypermethylation of the MGMT promoter is associated with longer survival in GBM patients treated with TMZ. Both DNA methylation-mediated silencing of the MGMT gene and high expression of ABC transporters could be reasons for ineffective TMZ treatment. Therefore, we explored the DNA methylation profile of the MGMT promoter region and investigated the expression level of GBM-implicated transporters in GBM cell lines. To evaluate the methylation status of the MGMT promoter region in untreated GBM cell lines, the COBRA assay was used (Figure 1). Almost complete methylation of the MGMT promoter was detected in U-118 MG (99%), SNB78 (98%), A172(98%), SF539 (95%), and SNB75 (86%) cell lines. The MGMT promoters of SNB19 (73%) and SF268 (70%) cell lines were moderately methylated, while the MGMT promoter of the H4 (14%) cell line was the least methylated.

### 2.2. Determination of Expression of ABC Transporters Using Quantitative Real-Time PCR (qPCR)

**Figure 2:**
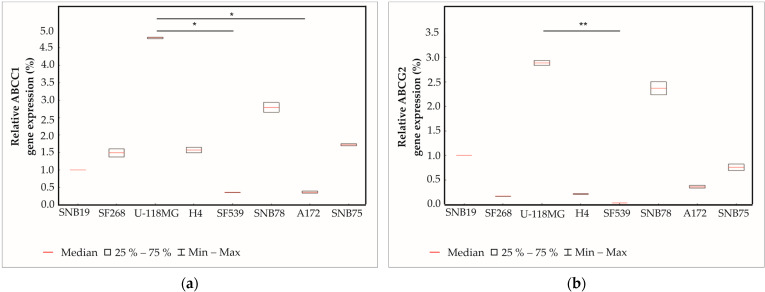
Box plots representing ABCC1 (**a**) and ABCG2 (**b**) gene expressions. Box plots have box boundaries ranging from the 25–75% percentiles, center line represents the median, and the whiskers illustrate the minimum and maximum of the data points. Significant results are presented as * *p* < 0.05, ** *p* < 0.01.

Since ABC transporters play an important role in the development of chemoresistance, the expressions of ABCC1 and ABCG2 transporters were studied in untreated GBM cell lines using qPCR (Figure 2). All cell lines expressed both ABCC1 (Figure 2a) and ABCG2 (Figure 2b) transporters. The highest expression of ABCC1 and ABCG2 was observed in U-118MG. The lowest expression of both transporters was detected in SF539 cells. The difference in expression was significant between U-118MG and SF539; however, there was no significant difference in expression compared to other cell lines.

### 2.3. Evaluation of the Sensitivity of GBM Cell Lines to TMZ and MLN4924

**Figure 3:**
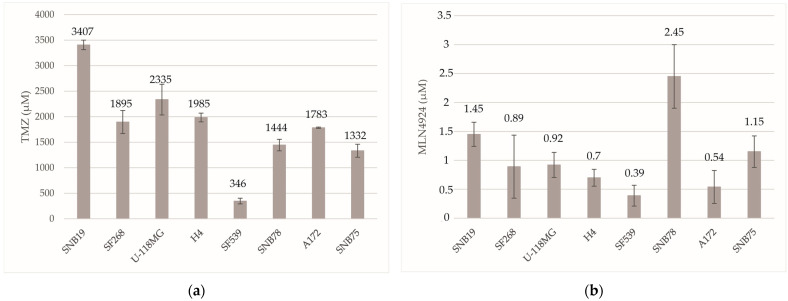
Comparison of IC_50_ values of different GBM cell lines treated with TMZ (**a**) or MLN4924 (**b**) for 48 h. GBM cell lines were treated for 48 h with different concentrations of TMZ or MLN4924 and the cell viability was measured by the ATP assay as described in Section 4. IC_50_ values were calculated based on the results of these experiments. Each bar represents the mean of three independent experiments, and error bars denote the standard deviation of the mean.

To determine the sensitivity of GBM cell lines to TMZ, cells were treated with different TMZ concentrations for 48 h and ATP content was measured using the CellTiter-Glo 2.0 Assay to determine cell viability, as described in Section 4. For the treatment, 1 μM, 1000 μM, and 3000 μM of TMZ were used. Relying on the results of previous experiments, these concentrations differentiate well between GBM cell lines based on their TMZ sensitivity. Data were normalized to the solvent containing control values for each cell line, and three independent experiments were performed. Based on the results of these experiments, the half-maximal inhibitory concentrations (IC_50_) of TMZ were calculated in Excel. IC_50_ values ranged from 346 to 3407 μM (Figure 3a), including the most TMZ-resistant SNB19 (3407 μM) and the most sensitive SF539 (346 μM) cells. Based on the IC_50_ values and the methylation status of the MGMT promoter, three cell lines (SNB19, H4, SF539) were selected for subsequential analysis. SNB19 cells are 9.8-fold more resistant to TMZ than SF539 cells. The H4 cell line is resistant to TMZ within limits; according to the IC_50_ values, it is 5.7-fold more resistant to TMZ than the SF539 cell line and 1.7-fold more sensitive to TMZ than the resistant SNB19 cells.

Targeted inhibition of NEDD8-activating enzyme E1 (NAE1) might increase the treatment efficacy of GBM alone or in combination with TMZ.

To determine the half-maximal inhibitory concentrations (IC_50_) of MLN4924, an inhibitor of the NEDD8-activating enzyme E1, GBM cell lines were treated with different concentrations of MLN4924 (0.01 μM, 0.1 μM, 1 μM) for 48 h and ATP content was measured to determine cell viability. Three independent experiments were performed and data were normalized to the solvent-containing controls for each cell line. IC_50_ concentrations were calculated relying on the results of the above treatments. IC_50_ values ranged from 0.39 to 2.45 μM (Figure 3b), including the most MLN4924-resistant SNB78 (2.45 μM) and the most sensitive SF539 (0.39 μM) cells. Based on TMZ sensitivity, three cell lines (SNB19, H4, SF539) were selected for further analysis, including the most TMZ-resistant SNB19, the moderately resistant H4, and the most sensitive SF539 cell line; therefore, only their MLN4924 sensitivity was compared below. The second most ML4924-resistant cell line is the TMZ-resistant SNB19, which is 3.7-fold more resistant to MLN4924 than SF539 cells. The H4 cell line is resistant to MLN4924 within limits; according to the IC_50_ values, it is 1.7 times more resistant to MLN4924 than the SF539 cell line and 2.0 times more sensitive to MLN4924 than the resistant SNB19 cells. Interestingly, the SF539 cell line is the most sensitive to both TMZ and MLN4924 treatment.

### 2.4. Effect of TMZ and MLN4924 Combination Treatments on Cell Viability

**Figure 4:**
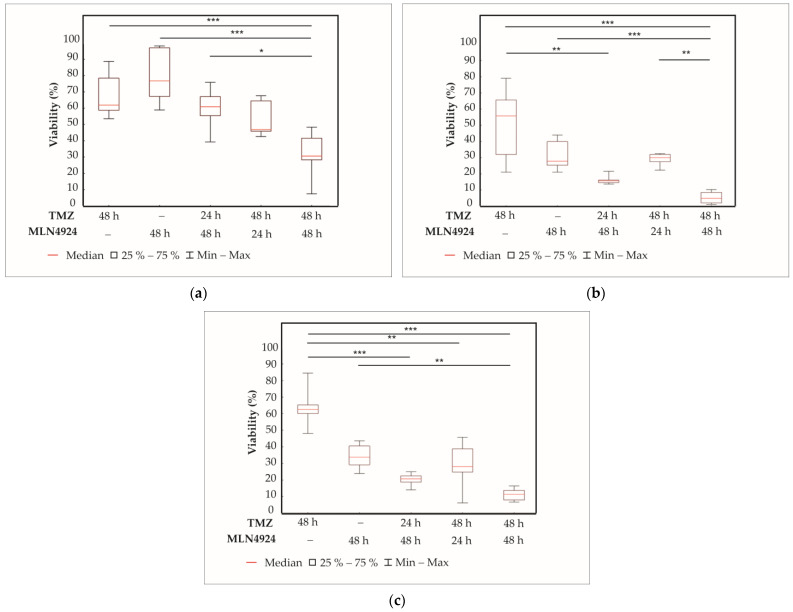
Box plots representing the effects of TMZ or MLN4924 treatments alone, or in combination in SNB19 (**a**), H4 (**b**), and SF539 (**c**) cell lines. The results of three independent experiments are shown. Box plots have box boundaries ranging from the 25–75% percentiles, the center line representing the median, and whiskers illustrating the minimum and maximum of the data points. Significant results are presented as * *p* < 0.05, ** *p* < 0.01, *** *p* < 0.001.

We next examined whether the neddylation inhibitor MLN4924 alters TMZ-induced cytotoxicity. Since MLN4924 induces DNA damage, it was hypothesized that MLN4924 may sensitize cells to TMZ in a way where DNA damage overwhelms DNA repair mechanisms. Previously selected cell lines (SNB19, H4, SF539) were treated with the appropriate IC_50_ concentrations of each drug alone or in different combinations for 48 h:24 h MLN4924 pretreatment followed by 24 h TMZ and MLN4924 treatments together (24 h TMZ + 48 h MLN4924); 24 h TMZ pretreatment followed by 24 h TMZ and MLN4924 treatments together (24 h MLN4924 + 48 h TMZ); 48 h TMZ and MLN4924 treatments together (48 h TMZ + 48 h MLN4924) (Figure 4). Cell viability was determined by the ATP assay. The result was normalized to the solvent containing the control values of each cell line.

Of the combination treatments, a 48 h combination of the two drugs (48 h TMZ + 48 h MLN4924) was the most effective. It resulted in significantly lower cell viability than either 48 h TMZ or 48 h MLN4924 treatments alone in all cell lines, and this effect was even pronounced in the highly resistant SNB19 cell line. The 24 h TMZ treatment in combination with 48 h MLN4924 treatment (24 h TMZ + 48 h MLN4924) was significantly more effective than the 48 h TMZ treatment alone in H4 (Figure 4b) and SF539 (Figure 4c) cell lines but not in the highly TMZ resistant SNB19 (Figure 4a) cell line. The 48 h TMZ treatment in combination with 24 h MLN4924 treatment (48 h TMZ + 24 h MLN4924) was significantly more effective than 48 h MLN4924 treatment alone in SNB19 but not in the other two cell lines and was not more effective than 48 h TMZ treatment alone.

### 2.5. Effect of TMZ and MLN4924 Combination Treatments on Apoptosis

**Figure 5:**
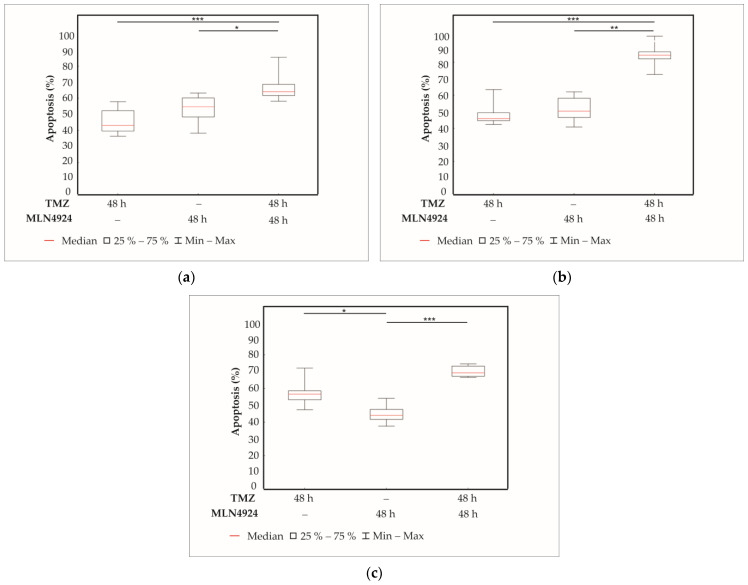
Analysis of the rate of apoptosis in SNB19 (**a**), H4 (**b**), and SF539 (**c**) cell lines. Cells were treated with the appropriate IC_50_ concentrations of TMZ and MLN4924 either alone or in combination (48 h TMZ + 48 h MLN4924) for 48 h. Cells were fixed and then stained with Hoechst 33342 dye. 200 cells per sample were counted in randomly chosen view fields using a fluorescence microscope. The rate of apoptosis was calculated as the percentage of the counted cells. At least 5 independent experiments were performed. Box plots have box boundaries ranging from the 25–75% percentiles, the center line represents the median, and the whiskers illustrate the minimum and maximum of the data points. Significant results are presented as * *p* < 0.05, ** *p* < 0.01, *** *p* < 0.001.

We next sought to determine whether the decrease in cell viability is caused by increased cell apoptosis. Previously selected GBM cell lines (SNB19, H4, SF539) were treated with TMZ and MLN4924 separately and with the most effective combination treatment (48 h TMZ + 48 h MLN4924). Cells were treated with the appropriate IC_50_ concentrations of each drug alone, or in combination for 48 h. Solvent-containing control and treated (48 h TMZ, 48 h MLN4924, and 48 h TMZ + 48 h MLN4924) samples were studied by fluorescence microscopy, and apoptotic cells were counted by nuclear morphology after staining with Hoechst 33342 dye (Figure 5).

In control samples, 4–8% of cells in each cell line were apoptotic. TMZ induced apoptosis in 45% of SNB19 cells (Figure 5a), 48% of H4 cells (Figure 5b), and 57% of cells in SF539 cells (Figure 5c). MLN4924 induced apoptosis in 53% of SNB19 cells, 51% of H4 cells, and 45% of SF539 cells. The combination treatments (48 h TMZ + 48 h MLN4924) were significantly more effective than TMZ or MLN4924 treatments alone in the TMZ-resistant SNB19 (67%) and H4 (84%). In the TMZ-sensitive SF539 cells, the combination treatment is significantly more effective than the MLN4924 treatment alone; however, there is no significant difference between the effect of combination treatment and TMZ treatment in the case of apoptosis.

### 2.6. Analysis of Expression of Proteins Involved in the Neddylation Pathway and TMZ Resistance

**Figure 6:**
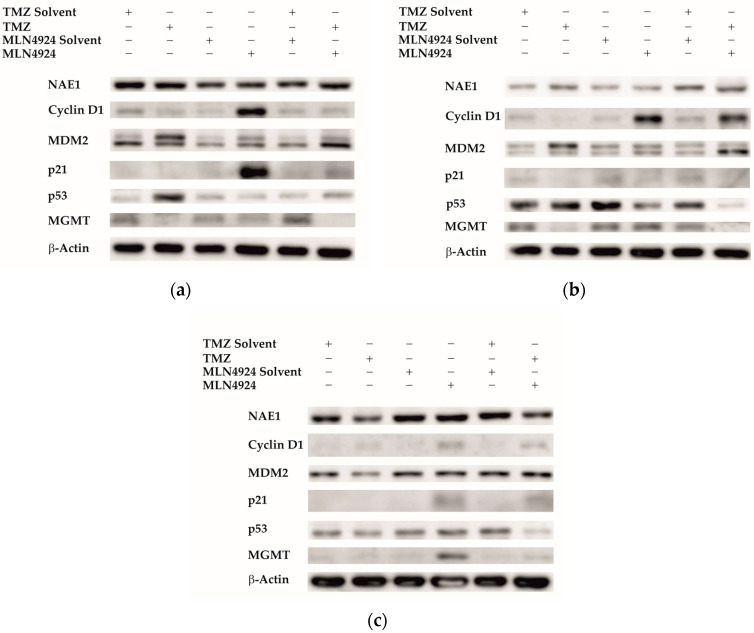
Western blot analysis of proteins involved in the neddylation pathway and TMZ-resistance in SNB19 (**a**), H4 (**b**), and SF539 cells (**c**). Cells were treated with the solvent of the agents or with the appropriate IC_50_ concentrations of TMZ and MLN4924 either alone or in combination (48 h TMZ + 48 h MLN4924) for 48 h. Western blot analysis was performed according to Section 4. β-actin was used as a loading control. At least three independent experiments were performed. Representative blots are shown.

Next, Western blot analysis was used to study the expression of proteins involved in the neddylation pathway and TMZ resistance. As the most effective combination treatment was 48 h TMZ combined with 48 h MLN4924 (48 h TMZ + 48 h MLN4924), this treatment combination was used in addition to single treatments (48 h TMZ, 48 h MLN4924) in SNB19 (Figure 6a), H4 (Figure 6b) and SF539 (Figure 6c) cells before analyzing different protein expressions. NAE1 expression levels were analyzed to evaluate the status of the neddylation pathway. All cell lines expressed the NAE1 protein, indicating that this pathway is active. The hallmark of NAE1 inhibition is the accumulation of CRL substrates (e.g., cyclin D1, Mdm2, and p21) and, therefore, these proteins were examined to demonstrate the effectiveness of MLN4924 treatment. All three MLN4924-treated cell lines expressed cyclin D1 relatively highly compared to their controls. In H4 and SF539 cells the cyclin D1 expression also increased due to the combination treatment. The relative amount of Mdm2 in SNB19 and H4 is higher as a result of the MLN4924 treatment; however, in SF539, there is no difference in Mdm2 expression in MLN4924-treated cells. In SNB19 and H4 cells, TMZ and combination treatment increased the Mdm2 expression compared to their controls. p21 accumulated in MLN4924-treated SNB19 and SF539 cells but not in MLN4924-treated H4 cells. Combination treatment increased p21 expression in SNB19 and SF539 cells compared to control samples. Taken together, 48 h of MLN4924 and combination treatment increased the amount of CRL substrate proteins (Mdm2, p21, cyclin D1). Since p53 is an important regulator of the cell cycle and repair processes and also a CRL substrate, we examined its expression in treated cells. The p53 protein is expressed in SNB19 cells; its expression is increased during TMZ and combination treatment and decreased during MLN4924 treatment. H4 control cells expressed p53, whose expression is increased during TMZ treatment and decreased during MLN4924 and combination treatment. In the SF539 cell line, p53 protein expression was increased during MLN4924 treatment and decreased during TMZ and combination treatment. The expression of MGMT protein in treated cells was also analyzed by Western blotting. The TMZ-resistant SNB19 and H4 cells express MGMT; however, its expression is decreased due to the combination treatment. The TMZ-sensitive SF539 expresses MGMT protein in a very low amount. Interestingly, its expression is induced during MLN4924 treatment.

### 2.7. Analysis of MGMT Protein Expression in Response to Combination Treatments by Immunocytochemistry

**Figure 7:**
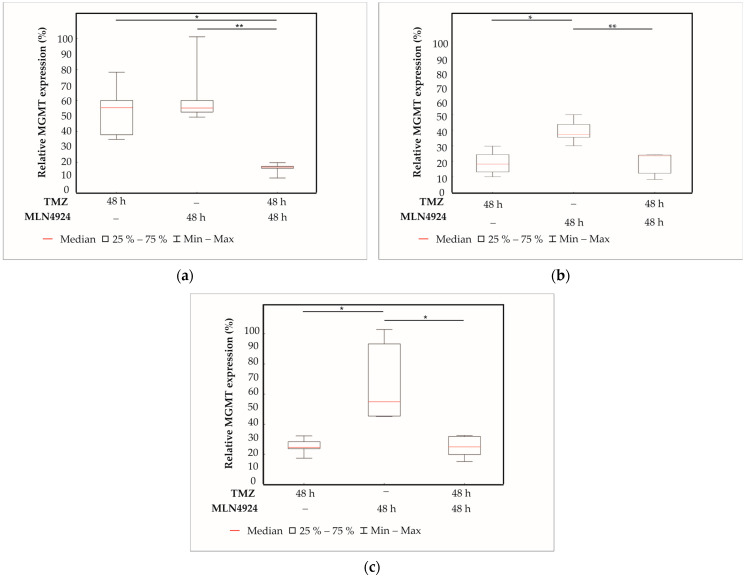
Box plots representing the quantitative analysis of the MGMT immunocytochemistry of TMZ or MLN4924 treatments alone, or in combination in SNB19 (**a**), H4 (**b**), and SF539 (**c**) cell lines. Data were normalized to the untreated control samples. The results of 3 independent experiments are shown. Box plots have box boundaries ranging from the 25–75% percentiles, the center line represents the median, and the whiskers illustrate the minimum and maximum of the data points. Significant results are presented as * *p* < 0.05, ** *p* < 0.01.

**Figure 8:**
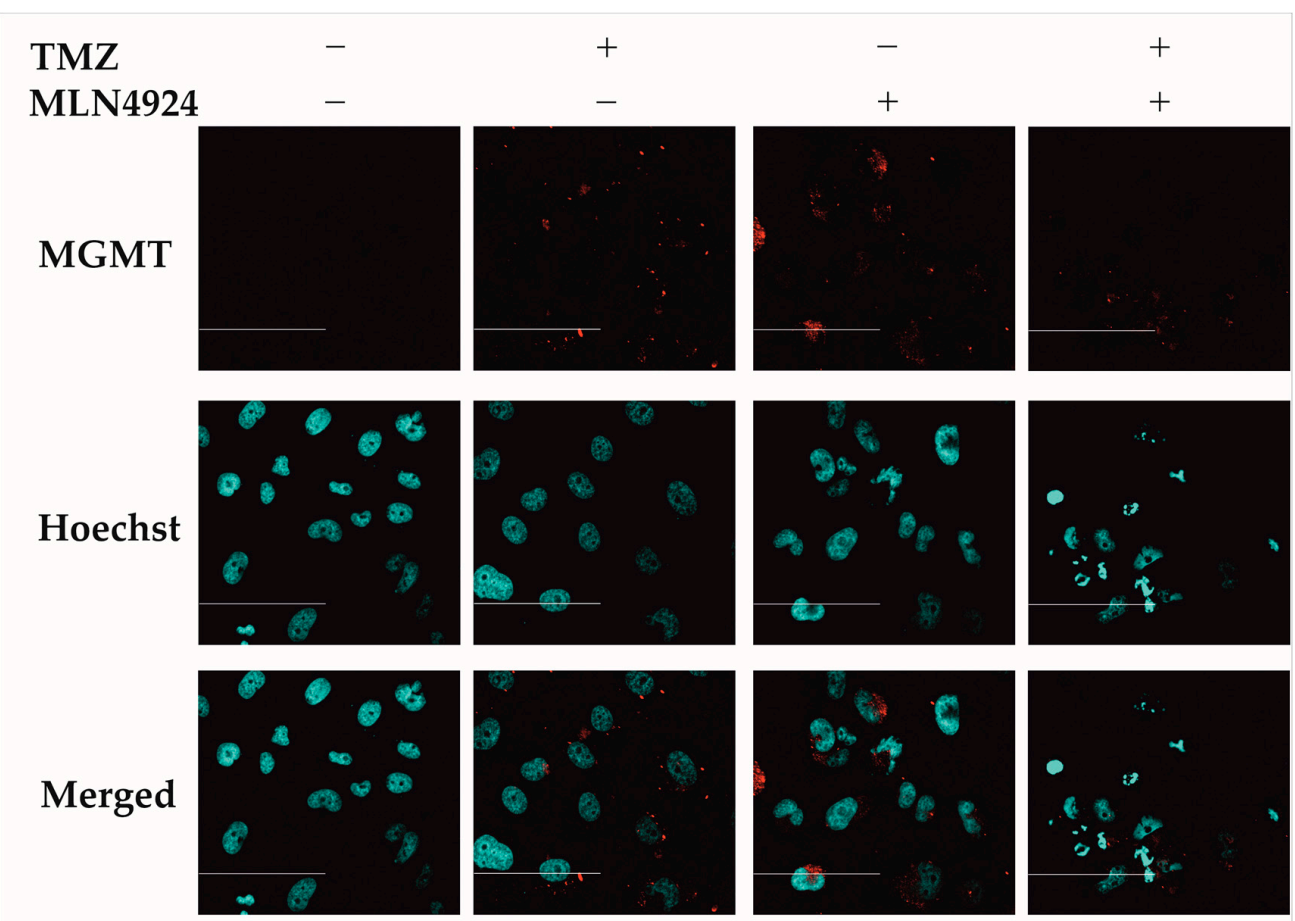
Detection of MGMT expression after single (48 h TMZ, 48 h MLN4924) and combination (48 h TMZ + 48 h MLN4924) treatments in SNB19 cells. Indirect immunocytochemistry was performed as described in Section 4. The red color indicates the presence of MGMT protein (upper panels), the blue color indicates cell nuclei stained by Hoechst 33342 (middle panels), and merge images are shown on the lower panels. The scalebar represents 100 μm. Representative images are shown.

To determine if 48 h TMZ, 48 h MLN4924, and combination (48 h TMZ + 48 h MLN4923) treatment alters MGMT protein expression in SNB19 (Figure 7a), H4 (Figure 7b), and SF539 (Figure 7c) cell lines, immunocytochemistry was performed using a specific MGMT antibody. Figure 7 shows the quantitative analysis of the immunocytochemistry performed with different treatments, and Figure 8 shows representative confocal microscopic images of the result of MGMT immunocytochemistry in SNB19 cells. In SNB19 cells, a strong MGMT expression was found in TMZ- and MLN4924-treated cells, however, the MGMT expression is significantly decreased due to the combination treatment. The expression of MGMT is significantly increased during MLN4924 treatment in H4 cells and significantly decreased compared to the combination treatment. There was no significant difference between the TMZ and combination treatment’s effects on MGMT expression. In SF539 cells, MGMT expression is the highest during MLN4924 treatment and it is significantly higher than that detected during 48 h TMZ or combination treatment.

## 3. Discussion

A number of signaling pathways, the expression level, and post-translational status of regulatory factors are significantly altered in GBM [20]. Due to this genetic and proteomic heterogeneity, the prognosis of GBM and the response of patients to therapy is mainly based on genetic variations and the epigenetic environment [21]. Methylation of the DNA repair enzyme MGMT gene promoter region leads to its epigenetic silencing that results in the reduction in DNA repair and increases chemosensitivity to TMZ [22]. We tested the sensitivity of several GBM cell lines to TMZ and selected three cell lines for further investigation. Although 73% of the MGMT promoter region was methylated in the SNB19 cell line, we detected expression of the MGMT protein, and this cell line showed the highest resistance to TMZ treatment. Of the cell lines tested, the MGMT promoter in the H4 cell line was the least methylated, and the expression of MGMT protein showed that this cell line was resistant to TMZ treatment. In the SF539 cell line, the MGMT promoter was highly methylated, MGMT protein expression was undetectable, and this cell line was the most sensitive to TMZ treatment. SF539 expressed ABCC1 and ABCG2 transporters in the lowest level, which can contribute to TMZ sensitivity. Previous studies have reported that the neddylation pathway is overactivated in several types of human cancers, including GBM, playing an important role in their development and progression and, therefore, the inhibition of this pathway could be utilized as a potential treatment for GBM [19]. We wanted to investigate whether we could overcome TMZ resistance by using a combination treatment with a drug that inhibits the overactive neddylation pathway in these cell lines. Inhibition of neddylation results in the accumulation of several intracellular proteins leading to DNA damage, induction of cell cycle arrest, and apoptosis [23]. Many proteins involved in the pathomechanism of GBM are neddylated; therefore, we used MLN4924, an inhibitor of the neddylation pathway, to determine the effect of the combination treatment. Combination treatment with TMZ + MLN4924 significantly reduced the viability compared to single TMZ treatment in all three selected cell lines, including the highly TMZ-resistant SNB19 cell line. Interestingly, the amount of MGMT protein is decreased in cells that are both treated with TMZ alone and in combination with MLN4924, indicating that this combination of drugs somehow intervenes in MGMT expression or degradation. The expression of MGMT in treated cells is inversely proportional to the expression of p53. Dora Bocangel et al. reported that MGMT expression is downregulated by p53 in human tumor cells [24]. Dimitris P. Xirodimas et al. reported that neddylation of p53 inhibits its transcriptional activity [25]. Therefore, inhibition of the overactivated neddylation pathway in GBM by MLN4924 may increase the transcriptional activity of p53, consequently downregulating MGMT. This effect was not pronounced in single MLN4924 treatments, however, it was in some cases of the combination treatments. MGMT protein expression is significantly decreased in combination-treated cells compared to temozolomide-treated cells in the highly TMZ-resistant SNB19 cell line. Due to this treatment p53 protein expression is increased in addition to decreased MGMT protein expression suggesting that TMZ in combination with MLN4924 is able to inhibit MGMT-mediated DNA repair through the enhancement of p53-mediated MGMT inhibition and could be the background of increased chemosensitivity. However, in the moderately TMZ-resistant H4 cells and the TMZ-sensitive SF539 cells, both p53 and MGMT protein expression is decreased according to combination treatment implying that another signaling pathway is involved in the TMZ sensitization in these cells.

Despite several novel therapeutic targets in the past decades, monotherapy has failed in clinical trials; therefore, combination therapy is becoming a key element of present-day antitumor therapy [26]. Other studies showed that NAE1 inhibitor MLN4924 may function as a novel chemosensitizer in various types of cancer such as esophageal squamous cell carcinoma, ovarian cancer, cervical carcinoma, and urothelial carcinoma [23,27,28,29]. A previous study demonstrated that MLN4924 in combination with cisplatin is an efficient strategy to target cisplatin resistance in ovarian cancer since MLN4924 enhances DNA damage and oxidative stress [28]. MLN4924 sensitizes leukemia cells to retinoic acid-induced apoptosis by inducing the accumulation of c-Jun and NOXA [30]. Liang Zhou et al., reported that MLN4924 in combination with belinostat reciprocally disables the DNA damage response in acute myelogenous leukemia [31]. Moreover, C Paiva et al., reported that MLN4924 in combination with alkylating agents (bendamustine, chlorambucil) sensitizes chronic lymphocytic leukemia B cells to alkylating agents through enhanced DNA damage and apoptosis [32].

Our study revealed that TMZ in combination with the NAE1 inhibitor MLN4924 can successfully decrease cell viability and induce apoptosis even in highly TMZ-resistant glioblastoma cell lines. However, the mechanism through which TMZ in combination with MLN4924 induces apoptosis requires further investigation. Further in vivo experiments are needed to confirm the effectiveness and clinical applicability of the TMZ and MLN4924 combination treatments.

## 4. Materials and Methods

### 4.1. Cells and Reagents

Human GBM cell lines H4 (HTB-148), U-118 MG, and A172 were obtained from the American Type Culture Collection (ATCC). SF539, SF268, SNB75, and SNB78 were obtained from National Cancer Institute (NCI), and SNB19 was obtained from Deutsche Sammlung von Mikroorganismen und Zellkulturen (DSMZ). H4, U-118 MG, A172, and SNB19 cell lines were cultured in Dulbecco’s Modified Eagle’s Medium (DMEM, Sigma-Aldrich Chemical Co., St. Louis, MO, USA), containing 4500 mg/mL glucose, 4 mM L-glutamine and 110 mg/mL pyruvate, supplemented with 10% fetal bovine serum (FBS) (Gibco, Carlsbad, CA, USA) at 37 °C and 5% CO_2_. SF539, SF268, SNB78, and SNB75 were cultured in RPMI-1640 (Sigma-Aldrich Chemical Co.) medium supplemented with 10% FBS at 37 °C and 5% CO_2_. Once cells reached 80% confluence, they were trypsinized and seeded onto a new Petri dish. Cells were used between passages 5 and 20 for the experiments. Temozolomide was purchased from Sigma-Aldrich Chemical Co. and dissolved in 10% Pluronic F-127 in 1× PBS (Sigma-Aldrich Chemical Co.). NAE1 inhibitor MLN4924 was purchased from Sigma-Aldrich Chemical Co. and dissolved in 10% DMSO (Sigma-Aldrich Chemical Co.) and 90% 20% Sulfobutylether-β-Cyclodextrin (SBE-β-CD) (Cyclolab Ltd., Budapest, Hungary) in 1× PBS.

### 4.2. Combined Bisulfite Restriction Analysis (COBRA)

For methylation analysis of the MGMT promoter, 10^5^ cells were plated from each cell line onto 60 mm plates (Greiner, Frickenhausen, Germany). For genomic DNA isolation and bisulfite conversion, the EZ DNA Methylation Direct Kit (Zymo Research, Irvin, CA, USA) was used according to the manufacturer’s instructions. The MGMT promoter region was amplified by PCR. The following primers were designed using the MethPrimer program (The Li Lab): forward primer 5′-GGGGTTTTTGATTAGGGGAG-3′ and reverse primer 5′-ACCTTTTCCTATCACAAAAATAATC-3′. The amplification was carried out in a 25 μL reaction mixture containing Maxima Hot Start Master Mix (2×) (Thermo Fisher Scientific, Waltham, MA, USA), 1 μM forward primer, 1 μM reverse primer, 1 ng bisulfite converted DNA template, and nuclease-free water. The PCR conditions were as follows: initial denaturation for 5 min at 95 °C, followed by 35 cycles of denaturation for 30 s at 95 °C, annealing for 30 s at 57 °C, and extension for 1 min at 72 °C and final extension for 5 min at 72 °C. After amplification, PCR products were digested at 37 °C overnight with Bsh1236I (BstUI) methylation-specific endonuclease enzyme (Thermo Fisher Scientific) specific for -CGCG- sequence, which digests alleles that were methylated prior to bisulfite treatment. After digestion, DNA fragments were separated in a 1.5% agarose gel stained with ethidium bromide. The percentage of MGMT promoter methylation was quantified using ImageJ software (version 1.53t) (NIH, Bethesda, MD, USA).

### 4.3. Quantitative Real-Time PCR (qRT-PCR)

Here, 10^6^ cells from each cell line were seeded on 100 mm cell culture plates. The next day, total RNA was isolated using a Direct-zol RNA Miniprep Plus Kit (Zymo Research) according to the manufacturer’s instructions. cDNA was synthesized by reverse transcribing 1 μg of total RNA using ProtoScript II First Strand cDNA Synthesis Kit (New England BioLabs, Ipswich, MA, USA) according to the manufacturer’s instructions. The program for the cDNA synthesis was the following: 25 °C 5 min 30 s, 42 °C 55 min, 48 °C 5 min, and 80 °C 5 min. qPCR reaction was performed using 1 μg cDNA and 10 pM/μL from the primers and by using a CFX RT-PCR instrument (BioRad, Hercules, CA, USA), using the following program: initial denaturation 95 °C 3 min, 40 cycles of 95 °C 10 s, 55 °C 30 s, 72 °C 1 min. Samples were amplified in duplicate and relative gene expression was analyzed using CFX Maestro Software (BioRad) and normalized to Beta-2 Microglobulin (B2MG). Primer sequences were designed using PrimerQuest software (version 2.2.3) and obtained from the IDT web page (Integrated DNA Technologies, Coralville, Iowa, USA) and were used to measure the expression of ABCG2 (forward primer 5′-CTTCGGCTTGCAACAACTATG-3′; reverse primer 5′-CCAGACACACCACGGATAAA-3′), ABCC1 (forward primer 5′-CGAGAACCAGAAGGCCTATTAC-3′; reverse primer 5′-ACAGGGCAGCAAACAGAA-3′) and B2MG (forward primer 5′-CAGCAAGGACTGGTCTTTCTAT-3′; reverse primer 5′-ACATGTCTCGATCCCACTTAAC-3′). The specificity of the PCR primers was supervised by a post-PCR melting curve analysis.

### 4.4. Cell Viability Assay

From SNB19, 2000 cells/well; from H4, 500 cells/well; and from SF539, 1000 cells/well were plated onto a poly-L-lysine coated white flat-bottom 96-well plate (Greiner) in triplicate. The next day, cells were treated with different concentrations of TMZ (1 μM, 1000 μM, 3000 μM) and MLN4924 (0.01 μM, 0.1 μM, 1 μM) for 48 h. At least three independent experiments were performed. For controls, identical amounts of either TMZ or MLN4924 solvents were added to the samples. The IC_50_ (half maximal inhibitory concentration) values of each drug were calculated according to the results of the aforementioned treatments in Excel. Then, cells were treated with these IC_50_ concentrations of TMZ and MLN4924 either alone or in combination (24 h TMZ + 48 h MLN4924; 24 h MLN4924 + 48 h TMZ; 48 h TMZ + 48 h MLN4924) for 48 h. Cell viability was measured using the CellTiter-Glo 2.0 Cell Viability Assay (Promega, Madison, WI, USA) according to the manufacturer’s instructions as described previously [33]. Luminescence was determined with a FLUOstar-OPTIMA V2.20 (BMG Labtech, Offenburg, Germany) luminometer.

### 4.5. Apoptosis Assay

From each cell line, 5 × 10^3^ cells/well were plated onto 96-well plates containing poly-L-lysine coated plastic coverslips (Greiner) in duplicates. The next day, cells were treated with the appropriate IC_50_ concentrations of TMZ or MLN4924 for 48 h either alone or in combination (48 h TMZ + 48 h MLN4924). After the treatment, cells were fixed in 4% paraformaldehyde (PFA) in 1× PBS. Cell nuclei were stained by Hoechst 33342 (Calbiochem, Darmstadt, Germany) fluorescent DNA dye in the final concentration of 0.5 μg/mL for 10 min. Samples were mounted onto coverslips using Vectashield (Vector Laboratories, Burlingame, CA, USA) anti-fading mounting medium. The percentage of apoptotic nuclei was determined by counting at least 200 cells/sample in randomly chosen view fields using an Olympus BX61 fluorescence microscope (Olympus, Center Valley, PA, USA). At least five independent experiments were performed.

### 4.6. Western Blot Analysis

From each cell line, 5 × 10^6^ cells were plated onto 100 mm plates (Greiner). The next day, cells were treated with the appropriate IC_50_ concentrations of TMZ or MLN4924 either alone or in combination (48 h TMZ + 48 h MLN4934) for 48 h. Cells were lysed in M-Per mammalian protein extraction buffer (Thermo Fisher Scientific) supplemented with protease inhibitor cocktail (Sigma-Aldrich Chemical Co.). Then, 40 μg of protein lysates were loaded onto 12% SDS-polyacrylamide gels and transferred onto PVDF membranes (Amersham, Buckinghamshire, UK). Membranes were blocked in 5% non-fat dry milk in 1× TBS-Tween (TBS-T) solution at room temperature for 2 h on a rotator and then incubated with the primary antibodies diluted in blocking buffer overnight at 4 °C on a rotator. The following primary antibodies were used: anti-NAE1 (final dilution 1:2000, Thermo Fisher Scientific), anti-cyclin D1 (1:500, Santa Cruz Biotechnology, Dallas, TX, USA), anti-Mdm2 (1:250, Santa Cruz Biotechnology, anti-p21 (1:500, Santa Cruz Biotechnology), anti-p53 (1:1000, Santa Cruz Biotechnology), anti-MGMT (1:200, Thermo Fisher Scientific). Anti-β-actin (1:1000, Cell Signaling Technology, Danvers, MA, USA) was used as a loading control. Membranes were washed five times for 5 min with 1× TBS-T and then incubated in species-specific horseradish peroxidase-conjugated secondary antibodies (Cell Signaling Technology) at 1:2000 final dilution for 2 h at room temperature on a rotator. Membranes were washed 5 times for 5 min in 1× TBS-T and the immunocomplexes were visualized by Pierce ECL Western Blotting Substrate (Thermo Fisher Scientific). The results were visualized using a G-box gel documentation system (Syngene, Cambridge, UK).

### 4.7. Immunocytochemistry

From each cell line, 5 × 10^3^ cells/well were plated onto 96-well plates containing poly-L-lysine coated plastic coverslips in duplicates (Greiner). The next day, cells were treated with the appropriate IC_50_ concentrations of TMZ and MLN4924 either alone or in combination (48 h TMZ + 48 h MLN4924) for 48 h. After the treatment, cells were fixed in 4% PFA in 1× PBS and incubated at 4 °C overnight. The next day, PFA was removed and cells were washed three times with 1× PBS for 5 min on a rotator. Cells were permeabilized in 0.5% Triton-X 100 (Sigma-Aldrich Chemical Co.) solution for 15 min at room temperature. Non-specific antibody binding sites were blocked by 3% bovine serum albumin (BSA, Sigma-Aldrich Chemical Co.) in 1× PBS at room temperature for 1 h on a rotator. Anti-MGMT primary antibody (Thermo Fisher Scientific) was added in a final dilution of 1:20 dissolved in 3% BSA in 1× PBS and then incubated overnight at 4 °C. The next day, samples were washed three times with 1× PBS for 5 min. Secondary Cy3-conjugated antibody (Jacksons ImmunoResearch Laboratories, West Grove, PA, USA) was added to the samples in a final dilution of 1:2000 dissolved in 3% BSA in 1× PBS and then cells were gently shaken overnight in the dark at 4 °C on a rotator. Non-specifically bound secondary antibodies were removed by washing the cells three times with 1× PBS. For negative control, cells were incubated only with the secondary antibody. Cell nuclei were stained by Hoechst 33342 (Calbiochem) fluorescent DNA dye in the final concentration of 0.5 μg/mL for 10 min. Samples were mounted onto coverslips using Vectashield (Vector Laboratories) anti-fading mounting medium and visualized by an Olympus FluoView 1000 confocal laser scanning fluorescence microscope (Olympus). The signal intensity for each cell was quantified using ImageJ software (version 1.53t).

### 4.8. Statistical Analysis

Statistical analysis was conducted using Statistica 10 software (Statsoft, Tulsa, OK, USA). Determination of normal distribution was conducted by the Shapiro–Wilk test. Statistical significance was confirmed by the Kruskal–Wallis test followed by Dunn post hoc test if significance was observed. *p* < 0.05 value was considered statistically significant.

## Data Availability

Not applicable.
